# Supplementary figures and images for: Shelters can negatively affect growth and welfare in lumpfish if feed is delivered continuously
Source: PeerJ. 2018 May 25;6:e4837. doi: 10.7717/peerj.4837 (PMC5971831; doi:10.7717/peerj.4837)

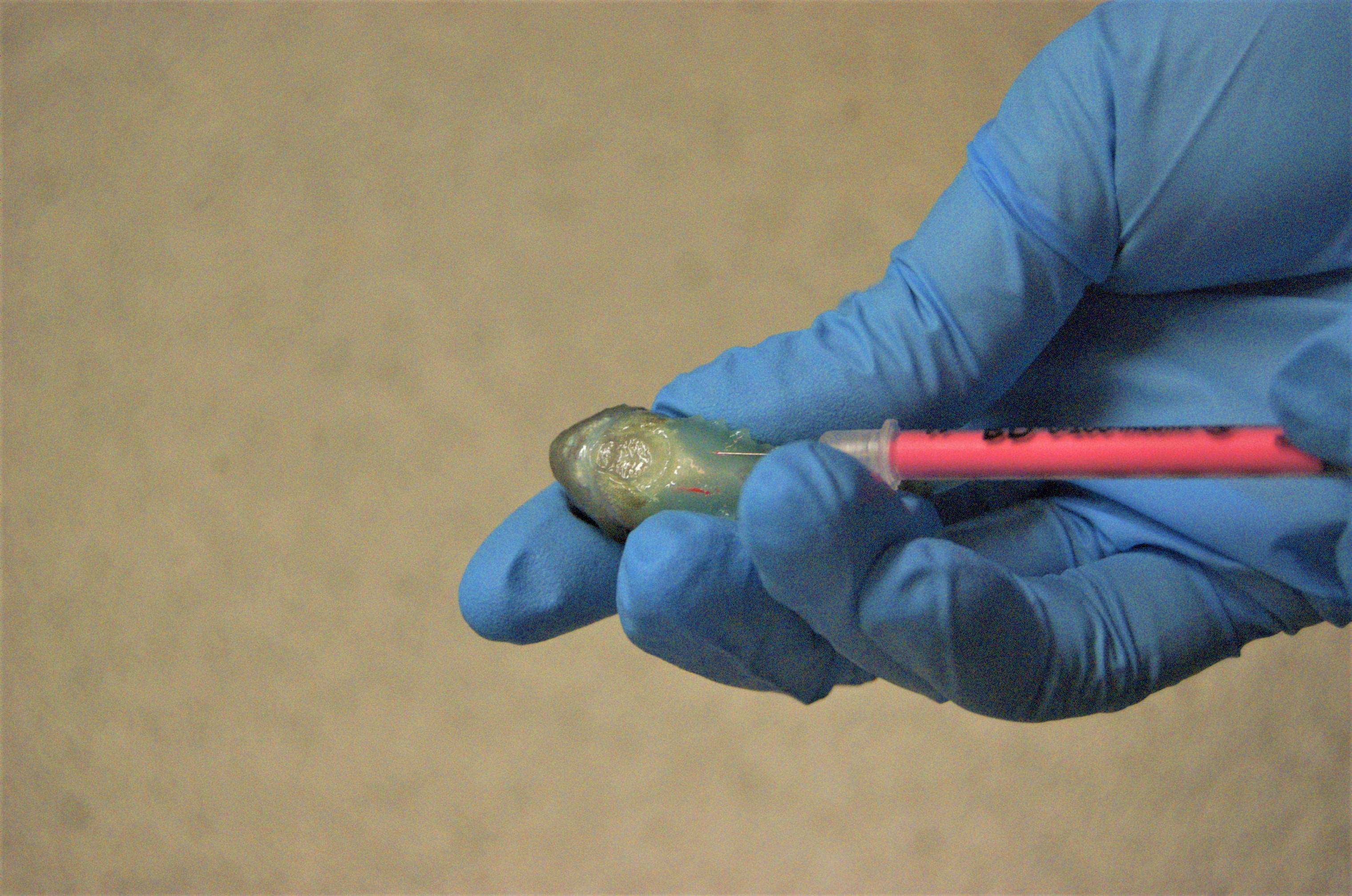

Supplement: Figure S1 — The tags are a liquid elastomer that is injected into the surface of the skin. In a few hours, the tags harden to a rubbery texture. Fish were colour-coded using a combination of two out of five colours for each fish in each tank. This tagging method works well for lumpfish because they do not have scales that obscure tags. However, due to potential dark pigmentation, care has to be taken to locate tags where minimal pigmentation is expected. Photo credit: Nakita E. Joensen. [file peerj-06-4837-s001.jpg]

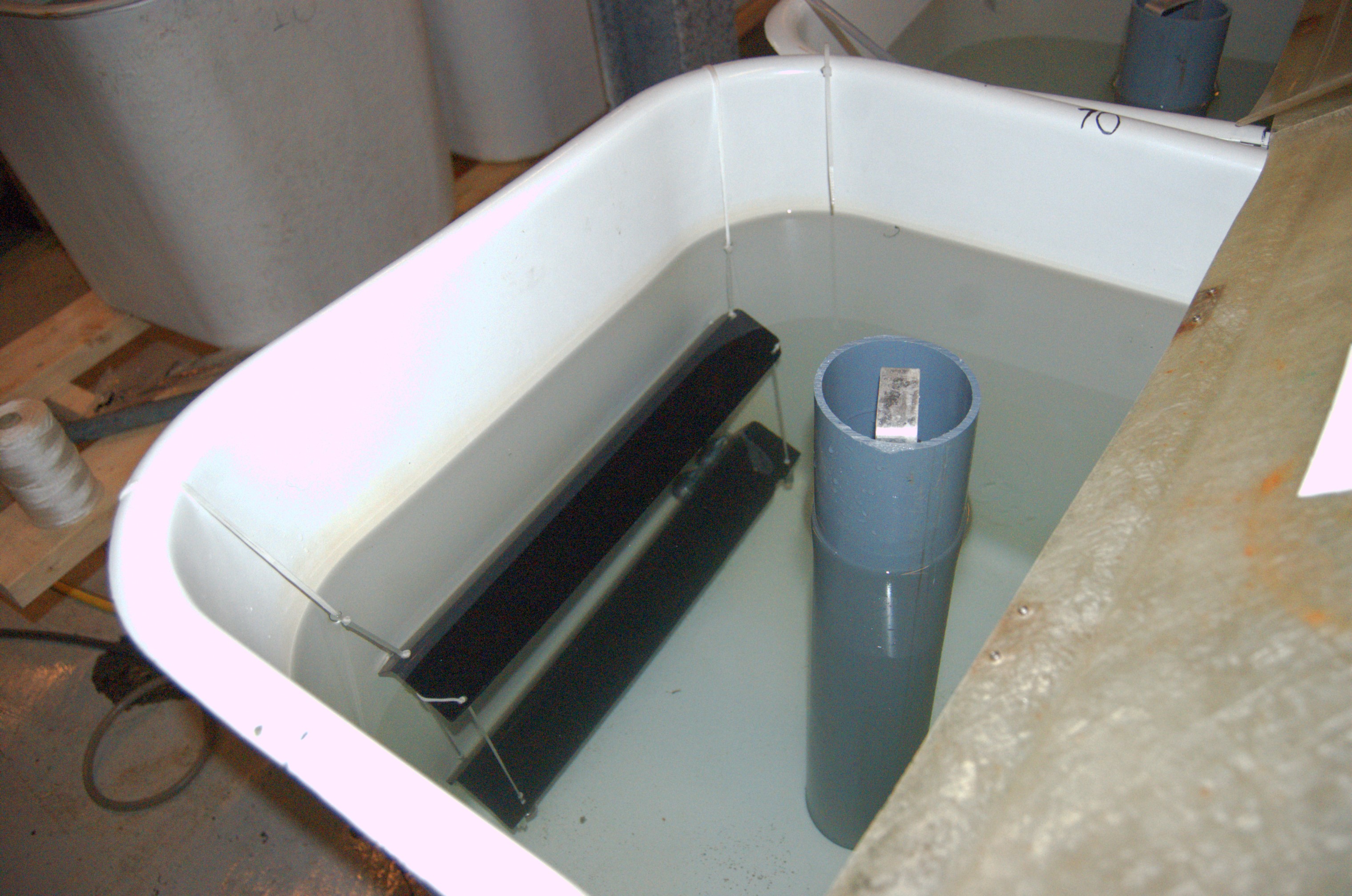

Supplement: Figure S2 — A tank with the two shelters hung as they were in all tanks with shelters. Upon careful inspection, it is possible to see some lumpfish sitting on the shelter. The remaining fish are most likely sitting underneath the shelter and so are obscured from view. Photo credit: Nakita E Joensen. [file peerj-06-4837-s002.jpg]
